# Clinical significance of plasma VEGF value in ischemic stroke - research for biomarkers in ischemic stroke (REBIOS) study

**DOI:** 10.1186/1471-2377-13-32

**Published:** 2013-04-08

**Authors:** Ryu Matsuo, Tetsuro Ago, Masahiro Kamouchi, Junya Kuroda, Takahiro Kuwashiro, Jun Hata, Hiroshi Sugimori, Kenji Fukuda, Seiji Gotoh, Noriko Makihara, Masayo Fukuhara, Hideto Awano, Tetsu Isomura, Kazuo Suzuki, Masahiro Yasaka, Yasushi Okada, Yutaka Kiyohara, Takanari Kitazono

**Affiliations:** 1 Department of Medicine and Clinical Science, Kyushu University, 3-1-1 Maidashi, higashi-ku, Fukuoka, 812-8582, Japan; 2 Environmental Medicine, Graduate School of Medical Sciences, Kyushu University, 3-1-1 Maidashi, higashi-ku, Fukuoka, Japan; 3 Department of Cerebrovascular Medicine, Clinical Research Institute, National Hospital Organization Kyushu Medical Center, 1-8-1 Jigyohama, chuo-ku, Fukuoka, Japan; 4 Department of Cerebrovascular Disease, St Mary’s Hospital, 422 tsubukuhonmachi, Kurume, Japan; 5 Molecuence Corporation, 1000 Kamoshidamachi, aoba-ku, Yokohama, Japan

**Keywords:** Brain infarction, Vascular endothelial growth factor (VEGF), National Institute of health stroke scale (NIHSS), Functional outcome, Modified rankin scale (mRS)

## Abstract

**Background:**

Vascular endothelial growth factor (VEGF) is a well-known molecule mediating neuronal survival and angiogenesis. However, its clinical significance in ischemic stroke is still controversial. The goal of this study was to examine the temporal profile of plasma VEGF value and its clinical significance in ischemic stroke with taking its subtypes into consideration.

**Methods:**

We prospectively enrolled 171 patients with ischemic stroke and age- and gender-matched healthy subjects. The stroke patients were divided into 4 subtypes: atherothrombotic infarction (ATBI, n = 34), lacunar infarction (LAC, n = 45), cardioembolic infarction (CE, n = 49) and other types (OT, n = 43). Plasma VEGF values were measured as a part of multiplex immunoassay (Human MAP v1.6) and we obtained clinical information at 5 time points (days 0, 3, 7, 14 and 90) after the stroke onset.

**Results:**

Plasma VEGF values were significantly higher in all stroke subtypes but OT than those in the controls throughout 90 days after stroke onset. There was no significant difference in the average VEGF values among ATBI, LAC, and CE. VEGF values were positively associated with neurological severity in CE patients, while a negative association was found in ATBI patients. After adjustment for possible confounding factors, plasma VEGF value was an independent predictor of poor functional outcome in CE patients.

**Conclusions:**

Although plasma VEGF value increases immediately after the stroke onset equally in all stroke subtypes, its significance in functional outcome may be different among the stroke subtypes.

## Background

Stroke is a leading cause of functional dependence and death [1]. It is important to prevent the occurrence of stroke and to perform immediate thrombolytic therapies when ischemic stroke occurs. The thrombolytic therapy using recombinant tissue plasminogen activator (rtPA) is becoming popular; however, the narrow therapeutic time window often limits the use of rtPA for patients with ischemic stroke [2]. Thus, we should make efforts to minimize neurological deterioration in the acute phase and to induce efficient neurological recovery in the sub-acute to chronic phase.

Vascular endothelial growth factor (VEGF), discovered in 1983 as a vascular permeability factor, is a key protein inducing angiogenesis in health and disease [3-5]. VEGF binds to the receptor tyrosine kinases VEGFR-1 (Flt-1) and VEGFR-2 (KDR/Flk-1) and mediates intracellular signaling leading to cell growth and survival in various cells. In the brain, VEGF is abundantly produced from neurons and vascular cells and acts on themselves, thereby playing an important role in mediating neuronal survival and angiogenesis [6]. Thus, one may expect that VEGF must exert beneficial effects in ischemic stroke. The expression of VEGF is transcriptionally upregulated by hypoxia-inducible factor in response to hypoxia. Consistently, in experimental ischemic stroke models, which mimic cardioembolic stroke in humans, the expression of VEGF and its receptor Flt-1 is upregulated in neurons and vascular cells in peri-infarct areas [7]. However, in these models, the suppression of the VEGF-Flt-1 signaling rather brings about beneficial effects on the brain [3]. It may be because VEGF induces endothelial proliferation and increases endothelial permeability leading to the breakdown of the blood–brain barrier [8,9]. Thus, the overall effect of VEGF on the brain in ischemic stroke is complicated and may be determined by ischemic strength, duration, and the presence of collateral circulation.

Brain infarction is classified into at least 4 different stroke subtypes, atherothrombotic infarction (ATBI), cardioembolic infarction (CE), lacunar infarction (LAC), and other types (OT). Ischemic strength, duration, and the presence of collateral circulation are different among the stroke subtypes. It has been reported that serum VEGF is increased in human stroke patients [10,11]. However, there have been no reports thus far examining in detail the difference of the clinical significance of VEGF among stroke subtypes. We hypothesized that due to the heterogeneity of ischemic stroke, the clinical significance of plasma VEGF may be different among stroke subtypes. In the present study, we examined the temporal profile of plasma VEGF value for 3 months after the stroke onset and the association of plasma VEGF with the neurological outcome in ischemic stroke, with taking its subtypes into consideration.

## Methods

### Ethics

The study protocol and all subsequent amendments are approved by the leading ethic committee of the Kyushu University Hospital (Kyushu University Institutional Review Board for Clinical Research, reference number #20-30, date of approval 9/26/2008) and the local ethics committees of the participating centers, National Hospital Organization Kyushu Medical Center (Kyushu Medical Center Institutional Review Board for Clinical Research, reference number # 07–38, date of approval 8/26/2007) and St Mary’s Hospital (St Mary’s Hospital Institutional Review Board for Clinical Research, date of approval 3/11/2008). The study is performed in accordance with the Declaration of Helsinki and its subsequent amendments, as well as the guidelines of Good Clinical Practice. The study is registered at the Fukuoka Stroke Registry (FSR), a multicenter observational study for acute brain infarction in Japan [12,13]. The registration number is #22-164.

### Study subjects

We recruited 171 patients with ischemic stroke who were hospitalized at Kyushu University Hospital, National Hospital Organization Kyushu Medical Center or St. Mary’s Hospital in Japan. On admission, the objectives, study design, risks and benefits were explained in detail to each patient or surrogate family members and written informed consent was obtained. Patients who consented to the study were prospectively enrolled and followed up to 3 months after the onset. Inclusion criteria were as follows: 1) ischemic stroke hospitalized within 24 hours after the onset, 2) definite diagnosis of stroke subtype. We excluded the patients who had severe complication, such as pneumonia or urinary tract infection, during the observational period.

An equal number of age- and gender-matched controls without a history of cardiovascular diseases, such as stroke, coronary heart diseases and atrial fibrillation, were enrolled as healthy subjects from the participants in the Hisayama Study, a worldwide well-known cohort study [14,15].

### Diagnosis of ischemic stroke and subtype

Stroke was defined as a sudden onset of focal neurological deficit persisting for more than 24 hours. The diagnosis of brain infarction was confirmed by brain imaging, including CT and MRI, in all patients. On the basis of the TOAST classification with minor modification, [16] we classified stroke patients into four categories (subtypes), i.e. atherothrombotic infarction (ATBI, n = 34), lacunar infarction (LAC, n = 45), cardioembolic infarction (CE, n = 49), and other type of brain infarction (OT, n = 43). We combined “Stroke of other determined etiology” and “stroke of undetermined etiology” into OT in this study.

### Data sampling

Peripheral venous blood samples were collected from patients at five time points after the onset, days 0 (on admission), 3, 7, 14, and 90. Blood samples were mixed in an EDTA-containing tube, and were centrifuged at 1,400 × g for 10 min at 4 degrees immediately after they were drawn. The resultant plasma samples were frozen at 80 degrees below zero within 10 minutes. They were stored for about two weeks until the measurement of VEGF. For healthy control subjects, blood samples were obtained at one point at the enrollment. Plasma VEGF values were measured using the Human Multi-Analyte Profile (MAP) v1.6 provided by Rules-Based Medicine, Inc. (RBM, TX, USA) [17]. A complete list of the analytes is available at http://www.myriadrbm.com/products-services/humanmap-services/.

### Risk factors

Hypertension was defined as systolic blood pressure ≥ 140 mmHg and/or diastolic pressure ≥ 90 mmHg or as current treatment with antihypertensive drugs during the chronic stage of stroke or at enrollment for control subjects. Diabetes mellitus was determined by either a 75 g oral glucose tolerance test according to the diagnostic criteria of the World Health Organization in 1998, [18] casual blood glucose levels (≥ 11.1 mmol/L (200 mg/dL)), or a medical history of diabetes. Dyslipidemia was defined as either a cholesterol level ≥ 5.7 mmol/L (220 mg/dL), a low-density lipoprotein-cholesterol level ≥ 3.62 mmol/L (140 mg/dL), a high-density lipoprotein-cholesterol level < 1.03 mmol/L (40 mg/dL) or current treatment with a cholesterol-lowering drug. Atrial fibrillation was diagnosed based on electrocardiographic findings or medical history. Smoking was defined as having a previous or current smoking habit. Alcohol intake was defined as having a previous or current consumption including occasional drinking.

### Evaluation of neurological severity and functional outcome

In analyses regarding neurological severity, patients were divided into tertiles (mild, moderate, and severe) in each stroke subtype, according to the National Institute of Health Stroke Scale (NIHSS). Functional outcome was evaluated by the modified Rankin Scale (mRS). Functional outcome was defined as poor (mRS score > 2) or good (mRS score ≤ 2). NIHSS was assessed at day 0 and 14, and mRS at day 14 and 90.

### Statistical analyses

JMP software ver.8.0 (SAS Institute, Cary, NC) was used to perform all statistical analyses. In univariate analyses, categorical variables were compared by the *χ*^2^ test, continuous variables by an unpaired Student *t* test or multiple comparisons as appropriate. In multivariate analyses, we performed logistic regression analyses with adjustment for confounding factors. P < 0.05 was considered significant.

## Results

### Background characteristics of control and stroke patients

Background characteristics of control subjects and stroke patients are summarized in Table 1 and Additional file 1: Table S1. The prevalence of hypertension, diabetes mellitus, atrial fibrillation and smoking were significantly higher in the stroke patients than the control. In contrast, alcohol intake was significantly higher in the control.

**Table 1:** Background characteristics of controls and stroke cases

	**Controls**	**Cases**	**p-Value**
	**n = 171**	**n = 171**	
Age, years, mean ± SD	68.1 ± 10.1	68.3 ± 10.1	0.84
Male, n (%)	115 (67.3)	115 (67.3)	1.00
Risk factors			
Hypertension, n (%)	72 (42.1)	132 (77.2)	<0.001
Dyslipidemia, n (%)	88 (51.5)	100 (58.5)	0.19
Diabetes, n (%)	11 (6.4)	54 (31.6)	<0.001
Atrial fibrillation, n (%)	0 (0)	59 (34.5)	<0.001
Smoking, n (%)	36 (21.1)	96 (56.1)	<0.001
Alcohol, n (%)	98 (57.3)	75 (43.9)	0.01

### Plasma VEGF values are increased immediately after stroke onset in all subtypes

**Figure 1:**
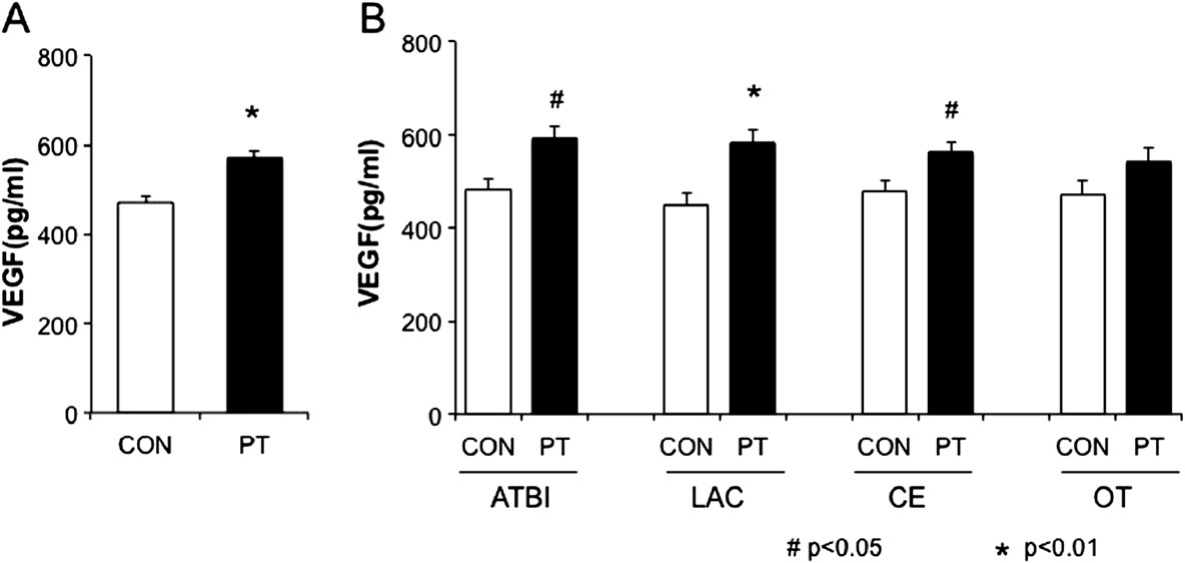
**Plasma VEGF values at day 0 after ischemic stroke.** Plasma VEGF levels at day 0 in stroke patients (PT, n = 171) and controls (CON, n = 171) (**A**), and those in each stroke subtype (**B**): atherothrombotic infarction (ATBI, n = 34), lacunar infarction (LAC, n = 45), cardioembolic infarction (CE, n = 49) and other type infarction (OT, n = 43). Data are expressed as mean ± SEM. *P < 0.01 vs. control, ^#^P < 0.05 vs. control.

Plasma VEGF values at day 0 were significantly higher in the patients (569 ± 13 pg/mL) than in the control (471 ± 13 pg/mL) (p < 0.001) (Figure 1A). A multivariate analysis adjusting for possible confounding factors demonstrated that plasma VEGF values were independently associated with brain infarction (OR 1.003 [1.001-1.005], p = 0.003). We investigated whether stroke-related risk factors or pretreatment affected plasma VEGF values. There was no association between the value of VEGF and stroke-related risk factors or pretreatment therapies, such as statins. For example, plasma VEGF values did not differ between in ischemic stroke patients with pretreatment of statin (n = 31, 611 ± 35 pg/mL) and those without (n = 140, 561 ± 17 pg/mL) (p = 0.20).

We classified stroke patients into 4 groups: ATBI (n = 34, 19.9%), LAC (n = 45, 26.3%), CE (n = 49, 28.7%), and OT (n = 43, 25.1%). Plasma VEGF values at day 0 were significantly higher in all stroke subtypes but OT (ATBI 593 ± 29 pg/mL, p = 0.016; LAC 584 ± 26 pg/mL, p = 0.003; CE 561 ± 24 pg/mL, p = 0.03; and OT 543 ± 30 pg/mL, p = 0.11) than those in the corresponding controls. There was no significant difference in the VEGF values among ATBI, LAC, and CE (Figure 1B).

### Temporal profile of plasma VEGF values in each stroke subtype

**Figure 2:**
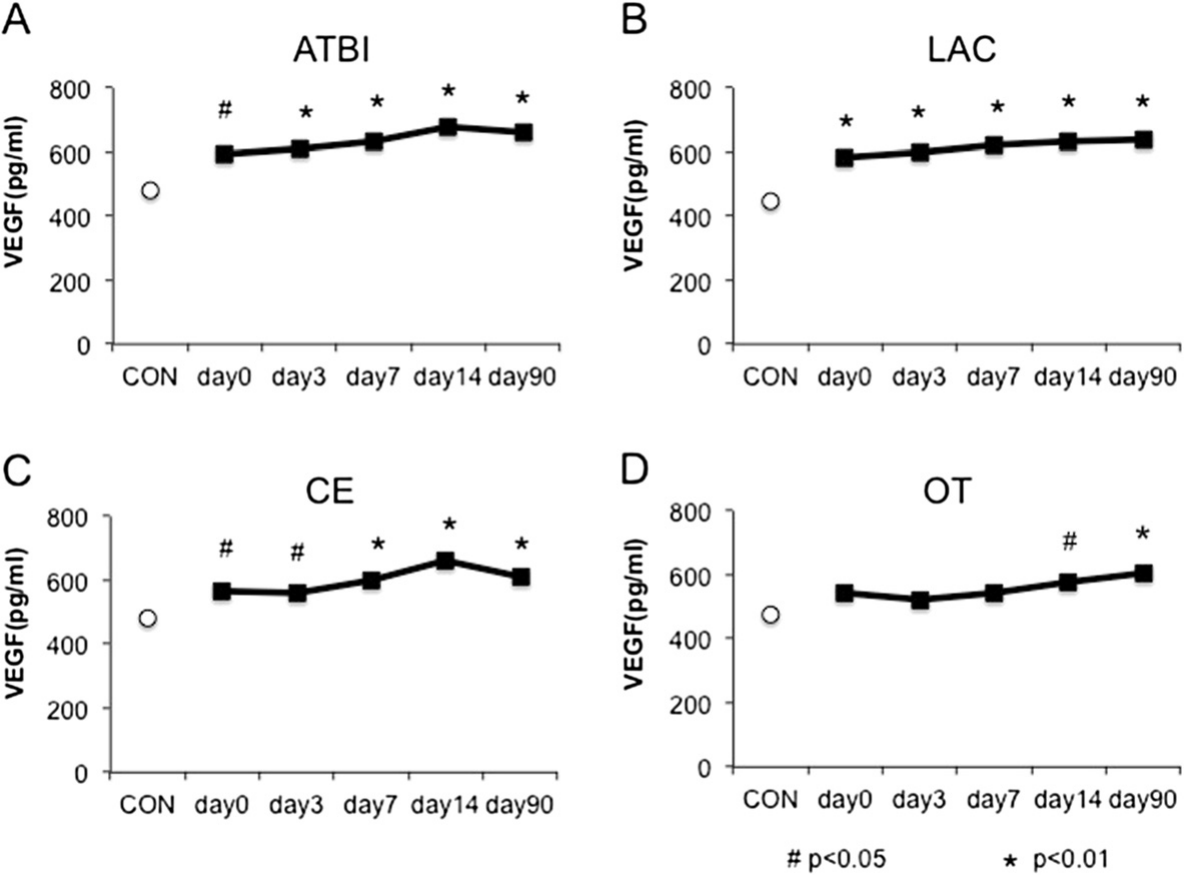
**Temporal profile of plasma VEGF levels in each stroke subtype from day 0 to day 90, A) ATBI, B) LAC, C) CE, D) OT.** Data are expressed as mean ± SEM. *P < 0.01 vs. control, ^#^P < 0.05 vs. control.

Temporal profile of plasma VEGF values in each stroke subtype from day 0 to day 90 is shown in Figure 2A (ATBI), 2B (LAC), 2C (CE) and 2D (OT). The increases in plasma VEGF values lasted for 90 days after the onset in all subtypes.

### Association between plasma VEGF value and neurological severity

**Figure 3:**
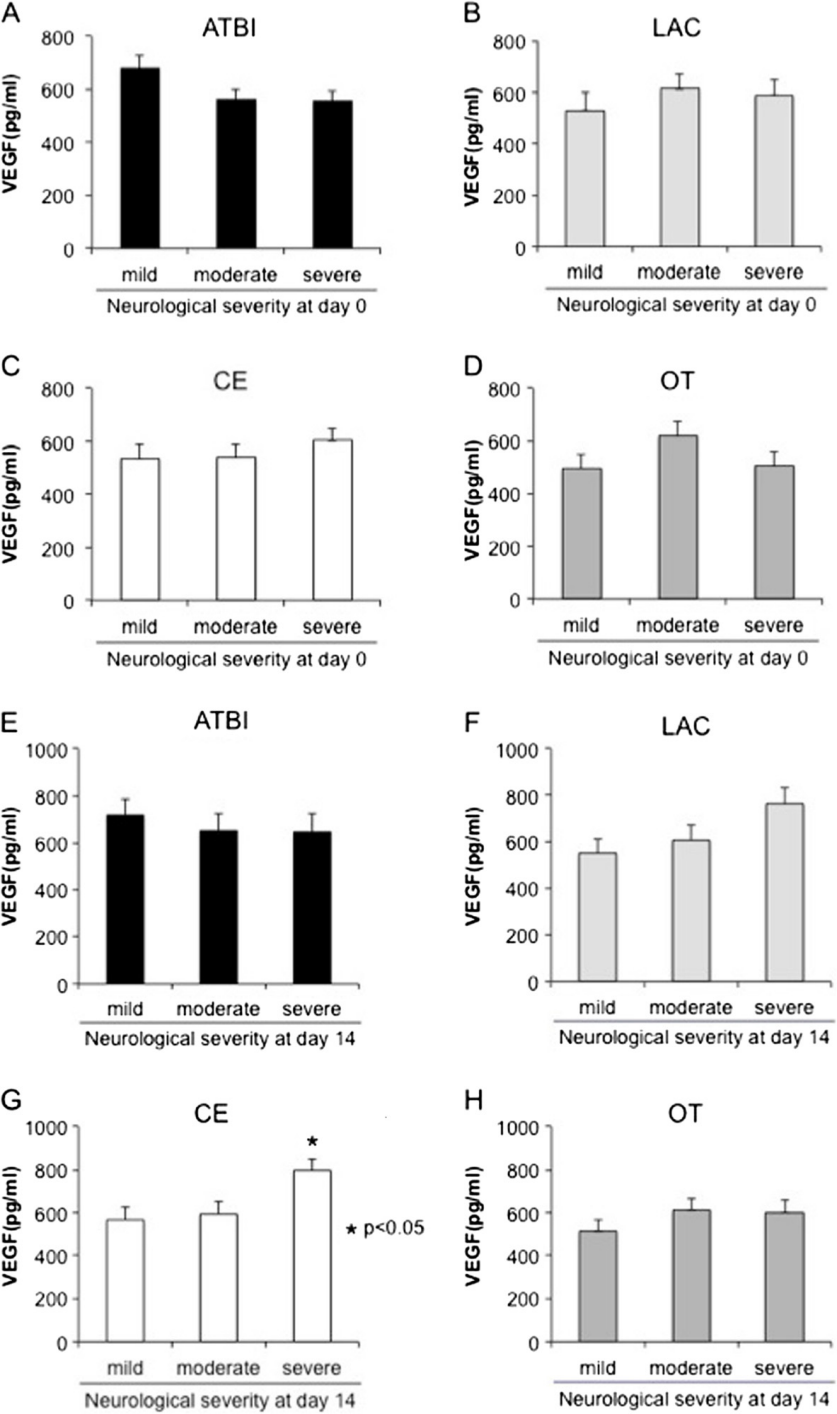
**Association between plasma VEGF values and neurological severity.** Association between VEGF values and neurological severity at day 0 in each stroke subtype, **A**) ATBI (mild 0–2; moderate 3–5; and severe 6–24); **B**) LAC (mild 0–1; moderate 2–3; and severe 4–9); **C**) CE (mild 0–3; moderate 4–9; and severe 10–25); **D**) OT (mild 0–2; moderate 3–4; and severe 5–31), and at day 14 in each stroke subtype, **E**) ATBI (mild 0–1; moderate 2–5; and severe 6–17); **F**) LAC (mild 0; moderate 1; and severe 2–6); **G**) CE (mild 0; moderate 1–3; and severe 4–24); **H**) OT (mild 0; moderate 1–2; and severe 3–24). Data are expressed as mean ± SEM. *P < 0.05 vs. mild group.

We next examined the association between plasma VEGF values and neurological severity on admission in each stroke subtype. The median NIHSS on admission was 4 (interquartile range (IQR): 2–8) in ATBI, 3 (IQR: 1–4) in LAC, 7 (IQR: 3–12) in CE and 4 (IQR: 2–6) in OT patients. We divided stroke patients into 3 groups in each subtype according to neurological severity as assessed by NIHSS at stroke onset. The cut-off values of NIHSS in each stroke subtype were as follows; in ATBI (mild 0–2; moderate 3–5; and severe 6–24); in LAC (mild 0–1; moderate 2–3; and severe 4–9); in CE (mild 0–3; moderate 4–9; and severe 10–25); and in OT (mild 0–2; moderate 3–4; and severe 5–31) (Figure 3 and Additional file 1: Table S2). Plasma VEGF values at day 0 were higher in the severe group in CE patients (Figure 3C), while they were higher in the mild group in ATBI patients (Figure 3A), although both failed to reach statistical significance. There was no association between VEGF values and neurological severity in LAC and OT patients (Figure 3B, Figure 3D). We also divided stroke patients into 3 groups at day 14 (Figure 3 and Additional file 1: Table S2). At day 14, the similar association was found in both CE (Figure 3G) and ATBI (Figure 3E), with statistical significance in CE (Figure 3G). There was no association between plasma VEGF values and neurological severity in LAC and OT patients (Figure 3F, Figure 3H).

### Association between plasma VEGF value and functional outcome

**Figure 4:**
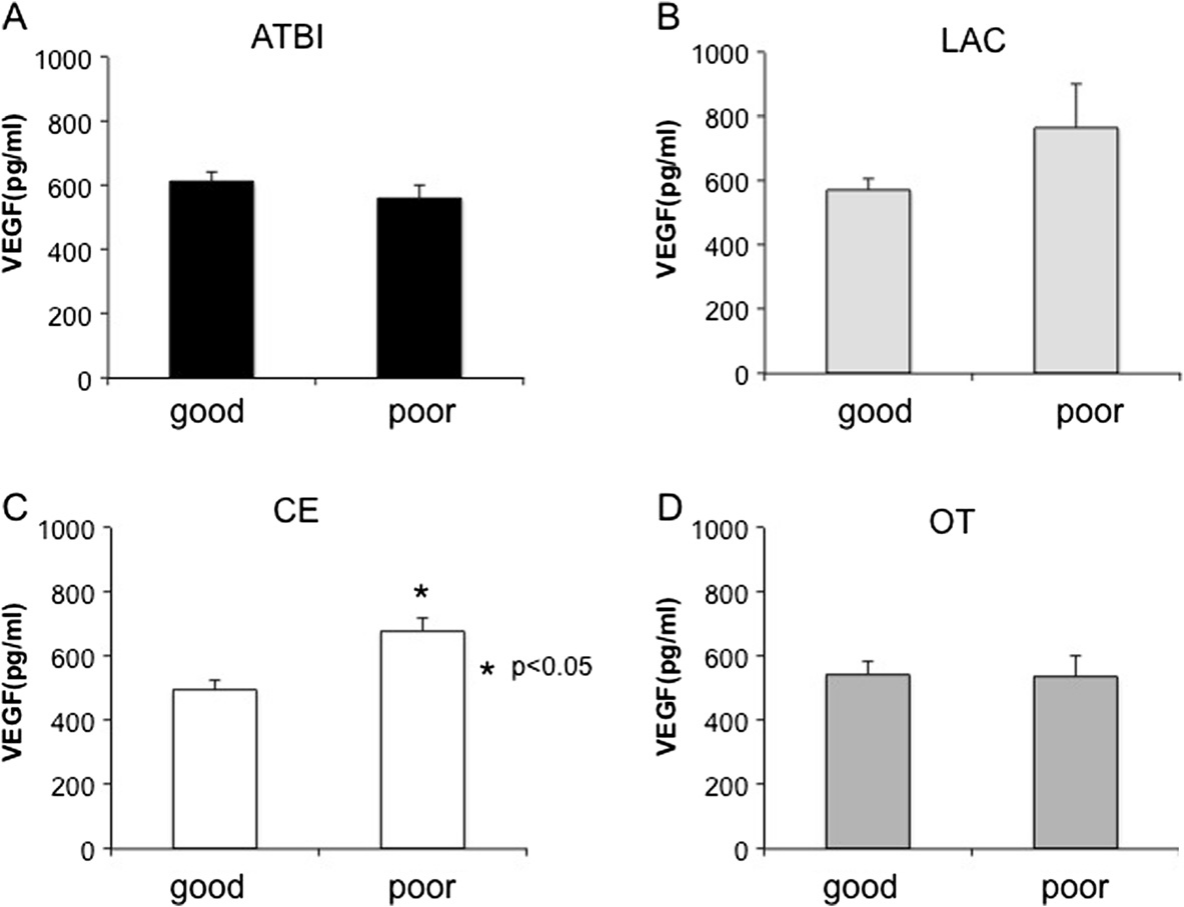
**Association between VEGF values and functional outcome.** VEGF values and functional outcome in each stroke subtype are shown in **A**) ATBI, **B**) LAC, **C**) CE, and **D**) OT. Data are expressed as mean ± SEM. *P < 0.05 vs. good group.

We further examined the impact of plasma VEGF values on functional outcome at day 90 in each stroke subtype. VEGF values at day 0 were significantly higher in the poor outcome group (mRS > 2; 681 ± 40 pg/mL) than in the good outcome group (mRS ≤ 2; 496 ± 31 pg/mL) in CE patients (p < 0.001; Figure 4C). In contrast, VEGF values tended to be higher in the good outcome group (619 ± 32 pg/mL) than in the poor outcome group (553 ± 43 pg/mL) in ATBI patients (p = 0.23; Figure 4A). There was not significant association between VEGF values and functional outcome in LAC patients (good: 573 ± 36 pg/mL vs poor: 767 ± 134 pg/mL; p = 0.17; Figure 4B) and in OT patients (good: 544 ± 37 pg/mL vs poor: 537 ± 67 pg/mL; p = 0.92; Figure 4D).

**Figure 5:**
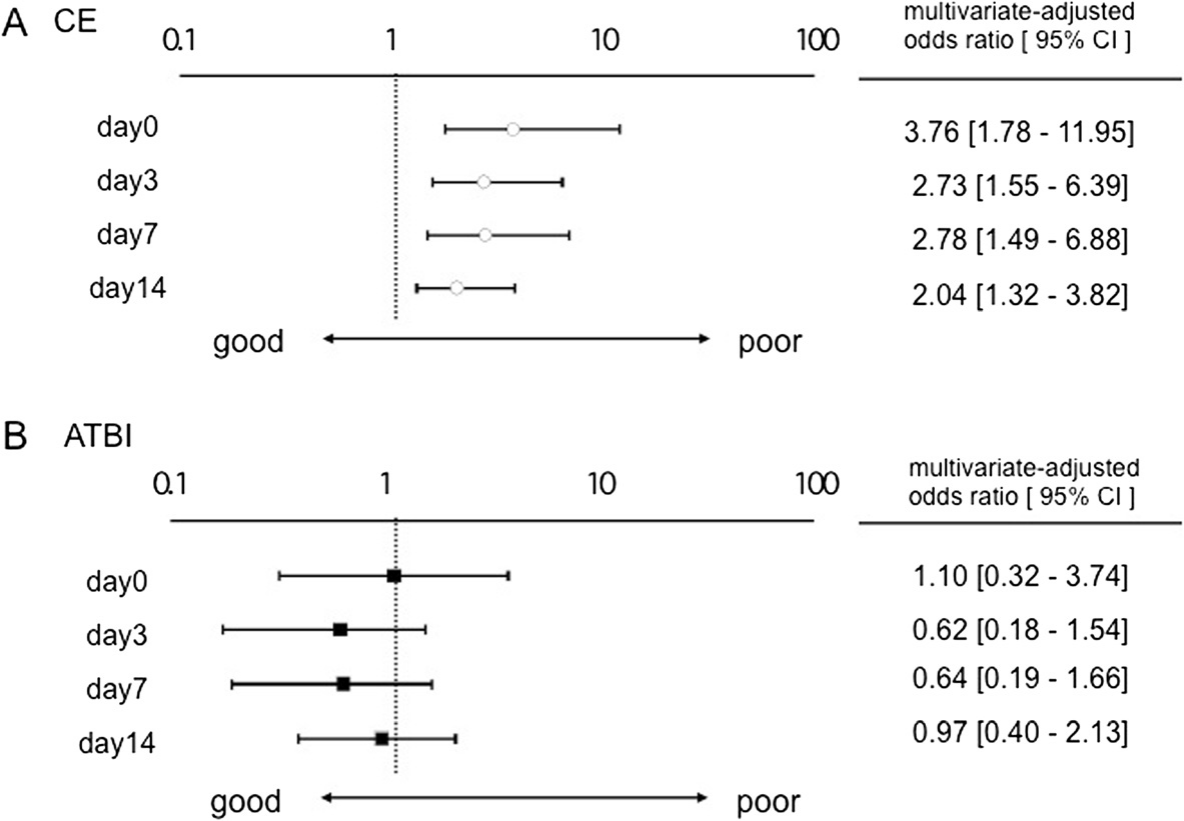
**Significance of plasma VEGF values in prediction of functional outcome.** Multivariate-adjusted odds ratio for functional outcome per 100 pg/mL increase in VEGF values at the indicated days after ischemic stroke in CE (**A**) and ATBI (**B**). The multivariate model included age, gender, hypertension, diabetes mellitus, neurological severity (NIHSS at day 0) and thrombolytic therapy. OR: odds ratio, CI: confidence interval.

Finally, we examined whether plasma VEGF value could be a predictor of functional outcome. We presented the data only of ATBI and CE, because there were only few patients with poor prognosis in LAC (n = 3) and there was no difference in plasma VEGF values between good prognosis group and poor one in OT (p = 0.92). A multivariate logistic regression analysis, adjusting for age, gender, hypertension, diabetes mellitus, neurological severity (NIHSS at day 0) and thrombolytic therapy revealed that the plasma VEGF value at day 0 could predict poor functional outcome at day 90 in CE patients with an odds ratio of 3.76 (95% confidence intervals: 1.78-11.95) per 100 pg/mL increase in VEGF (p < 0.001; Figure 5A). Similarly, higher VEGF values at day 3, 7 and 14 could predict poor functional outcome at day 90 in CE patients (Figure 5A). In contrast, higher VEGF values tended to predict good functional outcome in ATBI patients, although they did not reach statistical significance (Figure 5B).

## Discussion

In the present study, we demonstrated that 1) plasma VEGF values increased immediately after the onset, and the increase in VEGF values lasted for at least 90 days in all stroke subtypes, and 2) the clinical significance of plasma VEGF value in neurological severity and functional outcome was different among stroke subtypes. Higher plasma VEGF values may be a predictor of poor functional outcome in CE patients, while the opposite trend was found in ATBI patients.

Consistent with the present study, previous reports showed that serum VEGF value is increased in human stroke patients [10,11]. They demonstrated simply that the serum VEGF levels were correlated with infarct volume and neurological severity. The differences between our study and the previous one are that 1) plasma VEGF values increase in all stroke subtypes, 2) the increase of VEGF values lasts for 3 months in all subtypes, and 3) clinical significance of plasma VEGF values differs among stroke subtypes.

### Plasma VEGF value increases in all stroke subtypes

In the present study, plasma VEGF values were significantly higher in all stroke subtypes than those of the controls throughout the observation period (= 90 days) (Figure 2A-2D). Animal stroke models elucidated that the expression of VEGF is upregulated particularly in the ischemic penumbra [7,19,20]. Although we could not explain the reasons why plasma VEGF values were equally increased in all stroke subtypes, one possibility is that penumbral volumes may be similar even if necrotic volumes are different. Alternatively, humoral factors that are leaked or released from ischemic cells may diffuse into penumbral areas and equally induce the expression of VEGF regardless of infarct size. Another interesting finding of this study is that the increase in plasma VEGF value lasted for a longer period than we expected after the stroke onset in all stroke subtypes. A previous report showed that serum VEGF levels were higher in both the acute and chronic phases in the large vessel disease and only in the acute phase in the small vessel disease, compared with control. They concluded that increased serum VEGF levels might contribute to neurological improvement after 3 months [11]. The differences between our study and the previous one were that 1) we did not measure infarct volume, and 2) we used the plasma, but not serum, for VEGF measurement [17], although we do not know whether these differences affected the results.

Our observation suggests that the angiogenic response leading to neuronal regeneration continues at least for 3 months. Although hypoxia-inducible factors are well-characterized transcription factors that upregulate VEGF, [21] hypoxia itself may not continue for 3 months. Because VEGF plays a critical role in angiogenesis and neuronal regeneration after ischemic stroke, [22] further investigation is needed to elucidate the mechanisms underlying the prolonged production of VEGF irrespective of stroke subtypes.

### Clinical significance of VEGF value differs among stroke subtypes

Although plasma VEGF value was increased equally in all stroke subtypes, its clinical significance may be quite different among the subtypes. Plasma VEGF value was positively correlated with neurological severity in CE patients, whereas the opposite trend was observed in ATBI patients. LAC showed the similarity to CE rather than ATBI in terms of the association between VEGF values and functional outcome (Figure 3). A previous report showed that serum VEGF levels in the acute phase were significantly associated with the long-term prognosis of small and large vessel diseases, namely LAC and ATBI [11], which was partially consistent with our study.

In chronic and weak cerebral hypoxia in the patients with stenosis of the large cerebral arteries, VEGF may be increased already before stroke onset and function as an angiogenic [3] and neuroprotective molecule[6]. Thus, higher VEGF values would attenuate neuronal death and reduce infarct volume in ATBI. On the other hand, severe ischemia abruptly occurs without the absence of collateral circulation in CE, and thus VEGF values at the onset may simply reflect the intensity of ischemic damage in CE patients. In addition, a rapid increase in VEGF may increase the permeability of the blood–brain barrier, thereby worsening cerebral edema rather than functioning as a neuroprotective factor, as demonstrated in experimental stroke models [8,9]. Although VEGF can function as a neuroprotective and angiogenic molecule leading to neuronal regeneration, higher VEGF value in CE in the acute phase may rather associate with the severity of ischemic damage, thereby predicting poor functional outcome in CE patients. In terms of the abrupt ischemia and the absence of collateral circulation, LAC may be similar to CE rather than ATBI. Thus, the significance of plasma VEGF value in functional outcome in LAC may be similar to that of CE.

### Limitation

One of the limitations of this study is the small sample size. Further studies will be required to confirm the subtype-dependent clinical significance of VEGF suggested in the present study. Second limitation is the difference of risk factors between control and patients, although there was no association between VEGF values and risk factors. Third one is the lack of measurement of infarct volume in stroke patients. We used the NIHSS as an indicator of neurological severity instead of infarct volume.

## Conclusions

In conclusion, VEGF is increased in plasma immediately after the stroke onset in all stroke subtypes. The increase in plasma VEGF value continues for at least 90 days after the onset regardless of stroke subtype. Although it is established that VEGF plays important roles in neuronal survival and angiogenesis leading to neuronal regeneration, the clinical significance of plasma VEGF value may be different among the stroke subtypes.

## Abbreviations

ATBI: Atherothrombotic infarction; CE: Cardioembolic infarction; IQR: Interquartile range; LAC: Lacunar infarction; mRS: Modified rankin scale; NIHSS: National Institute of health stroke scale; OT: Other type of brain infarction; rtPA: Recombinant tissue plasminogen activator; VEGF: Vascular endothelial growth factor.

## Competing interests

The authors declare that they have no competing interests.

## Authors’ contributions

RM and TA conceived the study and drafted the manuscript. MK, JK, TK, HK, HA and TI participated in its coordination and helped to draft the manuscript. JH and SG participated in analysis and interpretation of data. KF, NM, MF, MY and YO participated in acquisition of data. TK, YK and KS designed the study. All authors read and approved the final manuscripts.

## Pre-publication history

The pre-publication history for this paper can be accessed here:

http://www.biomedcentral.com/1471-2377/13/32/prepub

## Supplementary Material

Additional file 1: Table S1 Background characteristics of controls and cases in each stroke subtype. **Table S2.** Association with VEGF values and neurological severity in each stroke subtype at day 0 (**A**) and at day 14 (**B**). Click here for file
