# Supplementary material for: Clinical significance of plasma VEGF value in ischemic stroke - research for biomarkers in ischemic stroke (REBIOS) study
Source: BMC Neurol. 2013 Apr 8;13:32. doi: 10.1186/1471-2377-13-32 (PMC3637234; doi:10.1186/1471-2377-13-32)
Supplement: Additional file 1: Table S1 — Background characteristics of controls and cases in each stroke subtype. Table S2. Association with VEGF values and neurological severity in each stroke subtype at day 0 (A) and at day 14 (B). [file 1471-2377-13-32-S1.pdf]

Supplement table 1. Background characteristics of controls and cases in each stroke subtype.

	Controls n=34	AT n=34	p-Value	Controls n=45	LAC n=45	p-Value	Controls n=49	CE n=49	p-Value	Controls n=43	OT n=43	p-Value
Age, years mean ± SD	70.3 ± 8.5	70.5 ± 8.1	0.95	67.2 ± 10.0	67.0 ± 9.5	0.95	70.7 ± 9.2	71.2 ± 9.6	0.79	64.4 ± 11.4	64.8 ± 11.5	0.89
Male, n (%)	23 (82.5)	23 (82.5)	1	29 (64.4)	29 (64.4)	1	35 (71.4)	35 (71.4)	1	23 (53.5)	23 (53.5)	1
Risk factors												
Hypertension, n (%)	18 (52.9)	30 (88.2)	0.001	17 (37.8)	35 (77.8)	<0.001	23 (46.9)	35 (71.4)	0.013	14 (32.6)	32 (74.4)	<0.001
Dyslipidemia, n (%)	17 (50.0)	27 (79.4)	0.011	22 (48.9)	27 (60.0)	0.29	26 (53.1)	21 (42.9)	0.31	23 (53.5)	25 (58.1)	0.66
Diabetes, n (%)	3 (8.8)	14 (41.2)	0.002	3 (6.7)	15 (33.3)	0.002	2 (4.1)	10 (20.4)	0.014	3 (7)	15 (34.9)	0.002
Atrial fibrillation, n (%)	0 (0)	10 (29.4)	<0.001	0 (0)	3 (6.7)	0.08	0 (0)	43 (87.8)	<0.001	0 (0)	3 (7)	0.08
Smoking, n (%)	10 (29.4)	21 (61.8)	0.007	11 (24.4)	25 (55.6)	0.003	6 (12.2)	27 (55.1)	<0.001	9 (20.9)	23 (53.5)	0.002
Alcohol, n (%)	22 (64.7)	14 (41.2)	0.052	26 (57.8)	17 (37.8)	0.058	27 (55.1)	21 (42.9)	0.23	23 (53.5)	23 (53.5)	1

Supplement table 2.

Association with VEGF values and neurological severity in each stroke subtype at day 0 (A) and at day 14 (B).

(A) Day 0

**ATBI**	**mild**	**moderate**	**severe**
NIHSS	0-2	3-5	6-24
n	9	13	12
VEGF(pg/ml), mean ± SD	682 ± 45	564 ± 38	557 ± 39
**LAC**	**mild**	**moderate**	**severe**
NIHSS	0-1	2-3	4-9
n	12	17	16
VEGF(pg/ml), mean ± SD	531 ± 68	614 ± 57	591 ±59
**CE**	**mild**	**moderate**	**severe**
NIHSS	0-3	4-9	10-25
n	13	16	20
VEGF(pg/ml), mean ± SD	536 ± 52	540 ± 47	603 ± 42
**OT**	**mild**	**moderate**	**severe**
NIHSS	0-2	3-4	5-31
n	13	15	15
VEGF(pg/ml), mean ± SD	495 ± 57	620 ± 53	507 ± 53

(B) Day 14

ATBI	mild	moderate	severe
NIHSS	0-1	2-5	6-17
n	12	11	8
VEGF(pg/ml), mean ± SD	718 ± 67	653 ± 70	645 ± 62
**LAC**	**mild**	**moderate**	**severe**
NIHSS	0	1	2-6
n	15	12	11
VEGF(pg/ml), mean ± SD	552 ± 58	609 ± 65	766 ± 68
**CE**	**mild**	**moderate**	**severe**
NIHSS	0	1-3	4-24
n	13	17	17
VEGF(pg/ml), mean ± SD	565 ± 65	596 ± 56	797 ± 56
**OT**	**mild**	**moderate**	**severe**
NIHSS	0	1-2	3-24
n	7	18	15
VEGF(pg/ml), mean ± SD	519 ± 74	552 ± 46	634 ± 50
